# Serum Interleukin-6 and CCL11/Eotaxin May Be Suitable Biomarkers for the Diagnosis of Chronic Nonbacterial Osteomyelitis

**DOI:** 10.3389/fped.2017.00256

**Published:** 2017-12-01

**Authors:** Sigrun Ruth Hofmann, Fanny Böttger, Ursula Range, Christian Lück, Henner Morbach, Hermann Joseph Girschick, Meinolf Suttorp, Christian Michael Hedrich

**Affiliations:** ^1^Department of Pediatrics, Medizinische Fakultät Carl Gustav Carus, Technische Universität Dresden, Dresden, Germany; ^2^Institute for Medical Informatics and Biometry, Medizinische Fakultät Carl Gustav Carus, Technische Universität Dresden, Dresden, Germany; ^3^Institute for Medical Microbiology, Medizinische Fakultät Carl Gustav Carus, Technische Universität Dresden, Dresden, Germany; ^4^University Children’s Hospital Würzburg, University of Würzburg, Würzburg, Germany; ^5^Children’s Hospital, Vivantes Klinikum im Friedrichshain, Berlin, Germany; ^6^Department of Women’s and Children’s Health, Institute of Translational Medicine, University of Liverpool, Liverpool, United Kingdom; ^7^Department of Paediatric Rheumatology, Alder Hey Children’s NHS Foundation Trust Hospital, Liverpool, United Kingdom

**Keywords:** chronic nonbacterial osteomyelitis, chronic recurrent multifocal osteomyelitis, inflammation, biomarker, autoinflammation, diagnosis

## Abstract

**Objectives:**

Chronic recurrent multifocal osteomyelitis (CRMO), the most severe form of chronic nonbacterial osteomyelitis (CNO), is an autoinflammatory bone disorder. In the absence of diagnostic criteria or biomarkers, CNO/CRMO remains a diagnosis of exclusion. The aim of this study was to identify biomarkers for diagnosing multifocal disease (CRMO).

**Study design:**

Sera from 71 pediatric CRMO patients, 11 patients with osteoarticular infections, 62 patients with juvenile idiopathic arthritis (JIA), 7 patients with para-infectious or reactive arthritis, and 43 patients with acute leukemia or lymphoma, as well as 59 healthy individuals were collected. Multiplex analysis of 18 inflammation- and/or bone remodeling-associated serum proteins was performed. Statistical analysis included univariate ANOVA, discriminant analysis, univariate receiver operating characteristic (ROC) analysis, and logistic regression analyses.

**Results:**

For 14 of 18 blood serum proteins, significant differences were determined between CRMO patients, at least one alternative diagnosis, or healthy controls. Multi-component discriminant analysis delivered five biomarkers (IL-6, CCL11/eotaxin, CCL5/RANTES, collagen Iα, sIL-2R) for the diagnosis of CRMO. ROC analysis allowed further reduction to a core set of 2 biomarkers (CCL11/eotaxin, IL-6) that are sufficient to discern between CRMO, healthy controls, and alternative diagnoses.

**Conclusion:**

Serum biomarkers CCL11/eotaxin and IL-6 differentiate between patients with CRMO, healthy controls, and alternative diagnoses (leukemia and lymphoma, osteoarticular infections, para-infectious arthritis, and JIA). Easily accessible biomarkers may aid in diagnosing CRMO. Further studies testing biomarkers in larger unrelated cohorts are warranted.

## Key Messages

- Serum CCL11/eotaxin and IL-6 allow differentiation between patients with chronic recurrent multifocal osteomyelitis (CRMO), healthy controls, and alternative diagnoses.
- Easily accessible biomarkers can aid in diagnosing CRMO.
- Further studies are needed to validate biomarkers in larger unrelated cohorts.

## Introduction

Chronic nonbacterial osteomyelitis (CNO) is an autoinflammatory bone disorder. Some patients exhibit monofocal and timely limited bone inflammation, while others develop chronically active or recurrent bone inflammation at multiple sites. This most severe presentation of CNO is referred to as CRMO (1–3). Despite intense efforts and some scientific progress over the recent years, the understanding of the pathophysiology of CNO/CRMO is still rudimentary. We recently demonstrated that monocytes from patients with severe and multifocal CRMO are characterized by impaired expression of the immune-regulatory cytokines interleukin (IL-)10 and IL-19, favoring the expression of pro-inflammatory cytokines (IL-1β, IL-6, IL-20, TNF-α). Though subsequent activation of osteoclasts has been suggested, the exact contribution of altered cytokine expression to bone inflammation and bone-loss remains somewhat unclear (3–7).

In the absence of widely accepted diagnostic criteria and disease biomarkers, CNO/CRMO remains a diagnosis of exclusion (3, 8, 9). Diagnosis may be delayed by unawareness of the disorder, variable clinical presentations, and sometimes rather mild symptoms. “Classical” inflammatory parameters, such as leukocyte counts, erythrocyte sedimentation rate (ESR), and C-reactive protein (CrP) are frequently normal or only mildly elevated. Furthermore, several autoimmune/inflammatory disorders, including oligoarticular juvenile idiopathic arthritis (JIA), share symptoms with CRMO. The absence of severe symptoms in individual cases, and lacking elevation of routine inflammation markers do not correlate with disease outcomes. Thus, the establishment of disease biomarkers for the diagnosis of CNO/CRMO is urgently needed (1–3, 8, 9).

Recently, we presented a set of serum biomarkers, including cytokines and chemokines that allowed discerning between CRMO, Crohn’s disease, and healthy individuals (10). However, we failed to discriminate between sera from patients with CRMO and JIA. Furthermore, other important differential diagnoses, including malignancies (acute leukemia, lymphoma) or osteoarticular infections, were missing (10). Here, we assessed 18 serum proteins to establish a set of serum biomarkers that allow discerning between healthy individuals, CRMO patients, and individuals with important differential diagnoses (JIA, osteoarticular infections, reactive arthritis, acute leukemia, and lymphoma).

## Materials and Methods

### Patients and Controls

Seventy-one pediatric patients with multifocal recurrent CNO, referred to as CRMO, 62 patients with JIA, 43 patients with either acute leukemia (*N* = 34) or lymphoma (*N* = 9), 11 patients with osteoarticular infections, and 7 individuals with reactive or para-infectious arthritis were included in the study. In the absence of evaluated diagnostic tools, CNO was defined using the clinical score from Jansson et al., additional laboratory findings, bone biopsies, and magnetic resonance imaging studies (3, 8, 9). All included patients developed multifocal CRMO. Serum samples from 20 out of the 71 CRMO patients and none of the other patients or controls enrolled in this study were also included in a previous analysis (10). Most samples were collected at University Children’s Hospital Dresden, some CRMO and JIA serum samples were from University Children’s Hospital Würzburg (10) (Data Supplement S1 in Supplementary Material). Samples were collected at diagnosis (from mostly treatment naïve patients, some JIA patients had received NSAIDs as needed) and stored at the bio-repository of the Institute of Microbiology, Faculty of Medicine Carl Gustav Carus, or University Children’s Hospital Würzburg until analysis. As normal controls, serum samples from 59 matched healthy individuals were collected at University Children’s Hospital Würzburg. The Ethics Committees of the University of Technology Dresden and the University of Würzburg approved collection and use of these samples. Serum samples from patients with other diagnoses were collected during routine care and stored in the bio-repository for potential additional diagnostic tests. Retrospective sample identification, collection and use of anonymized samples were approved by the Ethics Committe of the University of Technology Dresden. Since ethics approval required complete anonymization of all samples, retrospective analyses of JIA disease activity or long-term outcomes was impossible.

### Measurement of Serum Inflammatory Parameters

Blood samples were collected following standard procedures, and subjected to centrifugation. The liquid phase was separated from the pellets, transferred to cryo-tubes, and stored at −80°C until further use. According to manufacturer’s instructions, serum samples were diluted with LUMINEX buffer reagent (R&D Technologies) (1:1), and cytokines (IL-6, IL-10, IL-12p70, IL-18, IL-19, TNF-α, TRANCE/RANKL), chemokines (CCL2/MCP-1, CCL4/MIP-1β, CCL5/RANTES, CCL11/Eotaxin), the inflammatory protein S100A8, soluble cytokine receptors and antagonists (IL-1RA, sIL-2R), and bone metabolism markers (collagen Iα, osteopontin, osteoprotegerin, SPARC/osteonectin) were measured using a custom 18-plex (multiplex) assay (R&D Technologies) on the Luminex^®^ 200™ platform. Following this approach, all measured concentrations were within the detection range of the assay.

### Statistical Analysis

Serum protein levels that were below the detection range of our multiplex assay were set to “0.” Because of absent normal distribution of collected data, results are presented as dot blots, indicating mean values and SDs (inflammatory markers at time of diagnosis). For data analyses, JIA patients were subdivided in two groups (ANA positive oligoarticular JIA, and “other” JIA), since no statistical differences were determined between the following subgroups: polyarticular JIA (RF positive or negative), psoriatic arthritis, and enthesitis associated HLA-B27 positive JIA (ANOVA tests with *post hoc* Bonferroni adjustment; data not shown). Values from patients with para-infectious and reactive arthritis (“reactive”), or osteoarticular infections and Lyme arthritis (“infections”) were combined due to the lack of statistical differences between the individual diseases (data not shown).

For the comparison of serum inflammatory markers between controls and patients with CRMO, JIA, lymphoma or leukemia, osteoarticular infections, and reactive or para-infectious arthritis, data were analyzed using non-parametric univariate ANOVA tests (variance analysis) applying Bonferroni correction and pairwise comparison. Discriminant analysis was used to test biomarkers for their potential to discriminate between groups. Univariate receiver operating characteristic (ROC) analysis and logistic regression analysis were used to discriminate between groups based on single parameters. Statistical analyses were performed using SPSS software for windows (IBM, Chicago, IL, USA), version 23. Correlation analyses were performed using GraphPadPrism version 6.00 for Windows (GraphPad Software, La Jolla, CA, USA), and Pearson’s correlation coefficients were calculated.

## Results

### Serum Levels of Inflammation Markers

Based on data from a previous study (10), cytokines (IL-6, IL-12p70), chemokines (CCL2/MCP-1, CCL4/MIP-1β, CCL5/RANTES, CCL11/Eotaxin), and regulatory molecules (IL-1RA, sIL-2R) with differential serum concentrations in patients with CNO as compared to Crohn’s disease or JIA, and healthy individuals were included. Based on reports indicating that CNO is an autoinflammatory bone disorder with increased expression of monocyte-derived innate mediators of inflammation, the inflammatory protein S100A8, and bone metabolism markers (collagen Iα, osteopontin, osteoprotegerin, SPARC/osteonectin) were included (5, 7, 11, 12).

**Figure 1:**
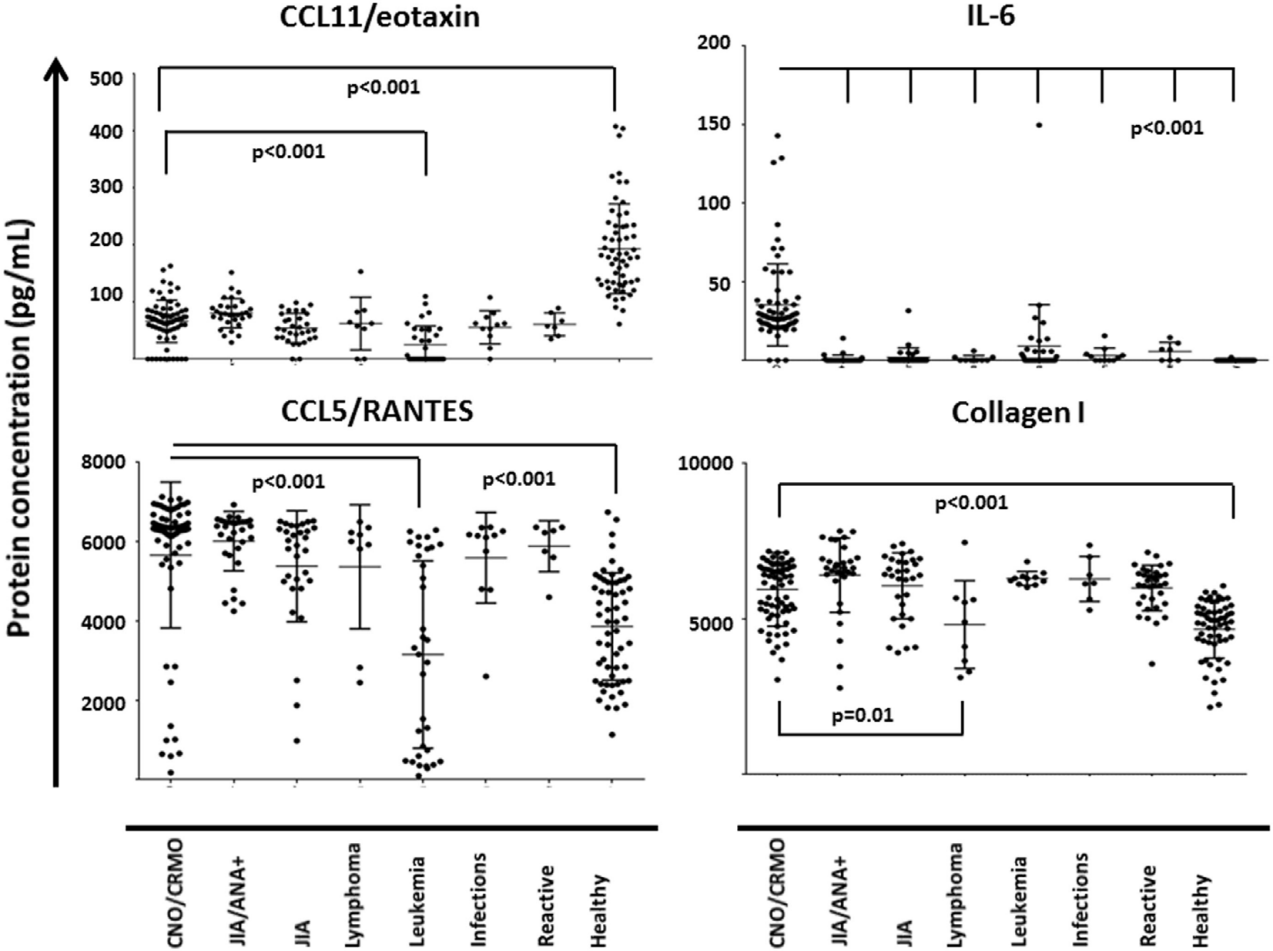
Serum inflammation markers in chronic recurrent multifocal osteomyelitis (CRMO) and alternative diagnoses. Serum inflammation markers were determined in samples from 71 CRMO patients, 11 patients with osteoarticular infections, 62 patients with various forms of juvenile idiopathic arthritis (JIA), 7 patients with para-infectious or reactive arthritis, and 43 patients with acute leukemia or lymphoma, as well as 59 healthy individuals using a custom multiplex assay (R&D Systems) on the Luminex^®^ 200™ platform. For the displayed inflammation markers, serum levels were detectable and significantly different for at least two of the groups. *p* values from univariate ANOVA tests applying Bonferroni correction and pairwise comparison are given. Adjusted *p* values of <0.05 were considered statistically significant.

For the comparison of serum inflammatory markers between healthy controls, patients with CRMO, and disease controls (JIA, acute leukemia or lymphoma, osteoarticular infections, and reactive or para-infectious arthritis), data were analyzed using non-parametric univariate ANOVA tests (variance analysis) applying *post hoc* Bonferroni correction and pairwise comparison (Data Supplement S2 in Supplementary Material; Figure S1 in Supplementary Material; Figure 1). The serum concentration of the chemokines and cytokines IL-12p70, IL-19, CCL2/MCP-1, and osteopontin were either below the detection range in all groups (IL-19) or did not show significant differences between normal controls and any of the disease groups and therefore are not included in figures (IL-12p70, osteopontin).

Patients with CRMO exhibited significantly increased serum levels of the cytokine IL-6 when compared to all other disease groups and healthy controls. All disease groups (including CNO/CRMO) were characterized by significantly reduced serum levels of CCL11/eotaxin as compared to healthy individuals. In the CRMO group, serum protein levels were above the levels in sera from healthy controls for the following parameters: CCL4/MIP-1β, CCL5/RANTES, IL-6, S100A8, sIL-2R, IL-1RA, IL-10, and collagen Iα (Figure S1 in Supplementary Material). When compared to sera from patients with ANA positive oligoarticular JIA, samples from CRMO patients had reduced levels of TRANCE/RANKL and CCL4/MIP-1β, but increased levels of IL-6. Serum samples from CRMO patients contained higher concentrations of CCL4/MIP-1β, CCL5/RANTES, CCL11/eotaxin, S100A8, and lower concentrations of IL-18, sIL-2R, and osteoprotegerin when compared to samples from patients with acute leukemia. When compared to sera from patients with lymphoma, samples from CRMO patients exhibited reduced levels of sIL-2R and increased levels of collagen Iα (Figure 1; Figure S1 and Data Supplement S2 in Supplementary Material).

### Discrimination between Groups

**Figure 2:**
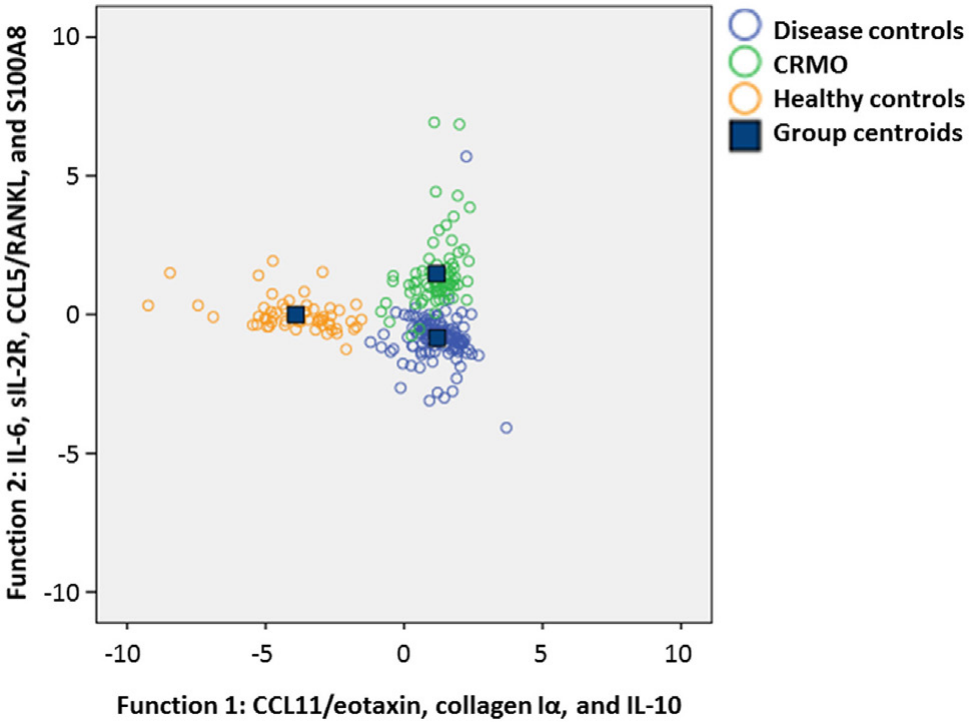
Diagnostic biomarkers for chronic recurrent multifocal osteomyelitis (CRMO). To test the applicability of serum proteins for discriminating between healthy individuals, patients with CRMO or alternative diagnoses, a discriminant analysis between the disease groups and healthy controls was performed. Standardized canonical discriminant coefficients were calculated and structure matrices were generated. Two functions were computer predicted. As indicated, function 1 correlates with CCL11/eotaxin, collagen Iα, and IL-10; function 2 correlates with IL-6, sIL-2R, CCL5/RANKL, and S100A8. Discrimination analyses with 2 functions, allow for differentiation among CRMO patients, healthy controls, and patients with alternative diagnoses.

To test whether the included serum parameters allow for discernment among healthy individuals, patients with alternative inflammatory conditions (JIA), infections (osteoarticular infections and Lyme disease), para-infectious disorders (reactive arthritis and para-infectious arthritis), malignancies (acute leukemia and lymphoma), and CRMO, discriminant analysis were performed (Figure 2). Standardized canonical discriminant coefficients were calculated and structure matrices were generated (Data Supplement S3 in Supplementary Material). Two functions were computer predicted: function 1 correlated with CCL11/eotaxin, collagen Iα, and IL-10; function 2 correlated with IL-6, sIL-2R, CCL5/RANKL, and S100A8 (Figure 2). Through discriminant analyses, differentiation between healthy individuals, CRMO patients, and patients with alternative diagnoses was possible. However, serum parameters did not discern between the individual differential diagnoses (JIA, lymphoma, leukemia, infectious, and reactive arthritis) (not shown).

**Figure 3:**
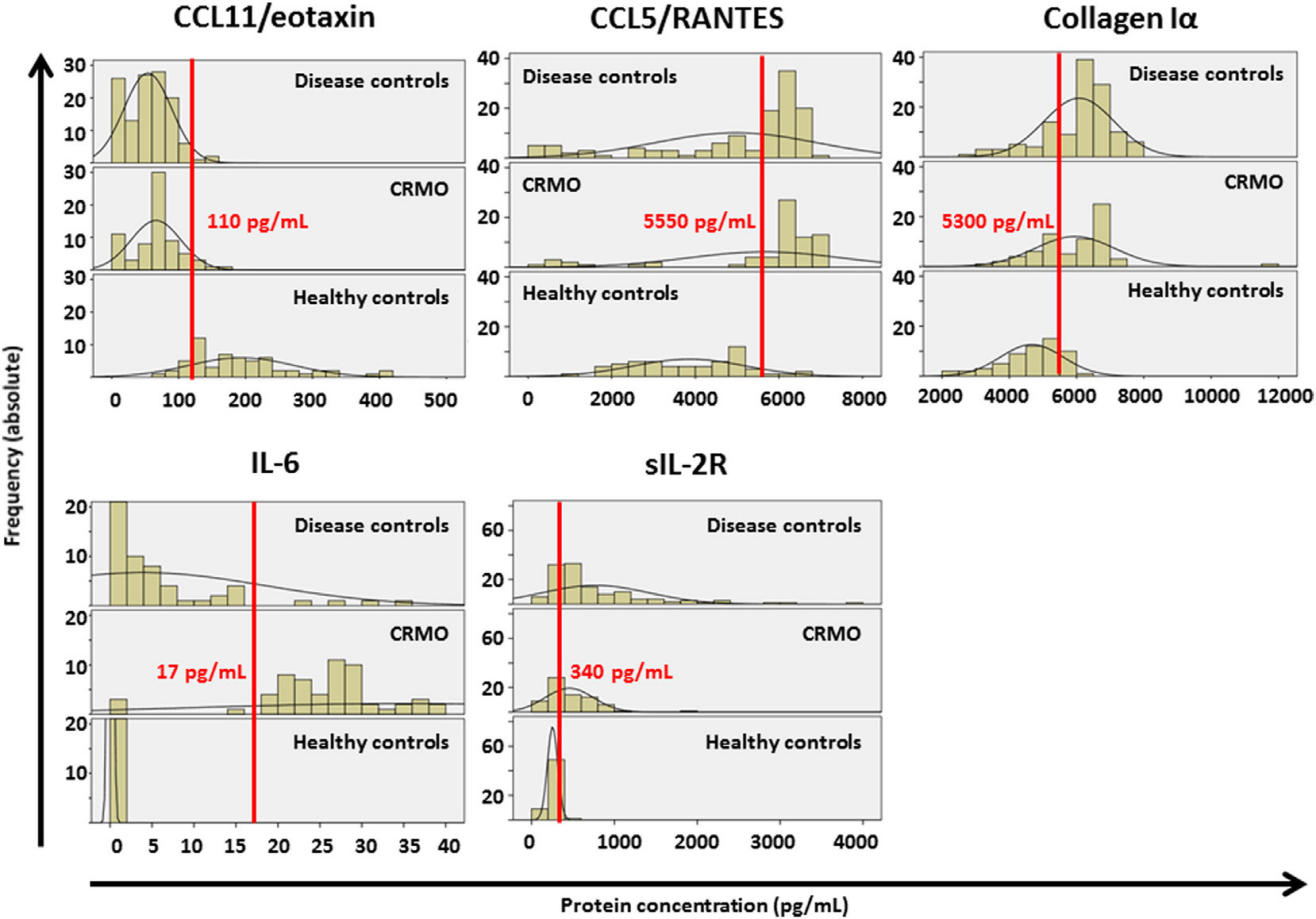
Cutoff value prediction. Histograms were generated, displaying the distribution of values from Figure 2 in all groups. Black lines reflect distribution curves (ideal curve that mean values and corresponding SDs would deliver). Only for CCL11/eotaxin and IL-6 minimal overlap between groups was determined, and cutoff values were identified (red lines, numbers indicate cutoff values in pg/mL).

To assess which parameters may be applicable as biomarkers for the diagnosis of CRMO and the exclusion of important alternative diagnoses, univariate ROC analysis was performed (Figure S2 in Supplementary Material). ROC analysis delivered CCL11/eotaxin as a promising candidate in the search for biomarkers differentiating between all included conditions and healthy controls (Figure S2A in Supplementary Material), and IL-6 as a potential biomarker discriminating between CRMO and all others (healthy controls and included inflammatory conditions) (Figure S2B in Supplementary Material). In addition to CCL11/eotaxin, ROC analysis suggested CCL5/RANTES, collagen Iα, and sIL-2Rα as potential diagnostic biomarkers. Thus, in a next step, histograms were generated, displaying the distribution of values in all groups (Figure 3). Only CCL11/eotaxin and IL-6 exhibited minimal overlap between groups (CCL11/eotaxin: CRMO and disease controls vs. healthy controls; IL-6: CRMO vs. disease and healthy controls), and cutoff values were identified. Starting with predicted cutoff points from univariate ROC analyses (CCL11/eotaxin: 102.44 pg/mL; IL-6: 14.976 pg/mL), we manually tested for the number of correct or incorrect predictions applying both parameters together. We predicted that CCL11/eotaxin levels >110 pg/mL may discriminate between healthy controls and inflammatory disorders, while IL-6 levels >17 pg/mL may discern between CRMO patients and all included controls (healthy and alternative diagnoses). Following this approach, prediction accuracy based on the selected values was tested (Data Supplement S4 in Supplementary Material). Two possible alternative approaches were compared: (i) discrimination between CRMO patients and individuals with alternative diagnoses starting with stratification based on IL-6 levels, followed by CCL11/eotaxin levels (Data Supplement S4A in Supplementary Material). Sensitivity of the approach was 93% and specificity was 97% (positive predictive value/PPV: 97%; negative predictive value/NPV: 95%). CRMO patients were discernable from healthy individuals with a sensitivity of 100% and a specificity of 98% (PPV: 98%; NPV: 100%). Lastly, CRMO patients were identified from all other samples (healthy plus disease controls) with a sensitivity of 94% and a specificity of 97% (PPV: 93%, NPV: 97%). (ii) Stratification based on CCL11/eotaxin levels, followed by IL-6 (Data Supplement S4B in Supplementary Material), delivered discrimination with a sensitivity of 100% and a specificity of 87% (PPV: 95%; NPV: 96%). CRMO patients were discernable from healthy individuals with a sensitivity of 93% and a specificity of 97% (PPV: 89%, NPV: 100%). Lastly, CRMO patients were identified from all other samples (healthy plus disease controls) with a sensitivity of 92% and a specificity of 94% (PPV: 84%, NPV: 97%).

**Figure 4:**
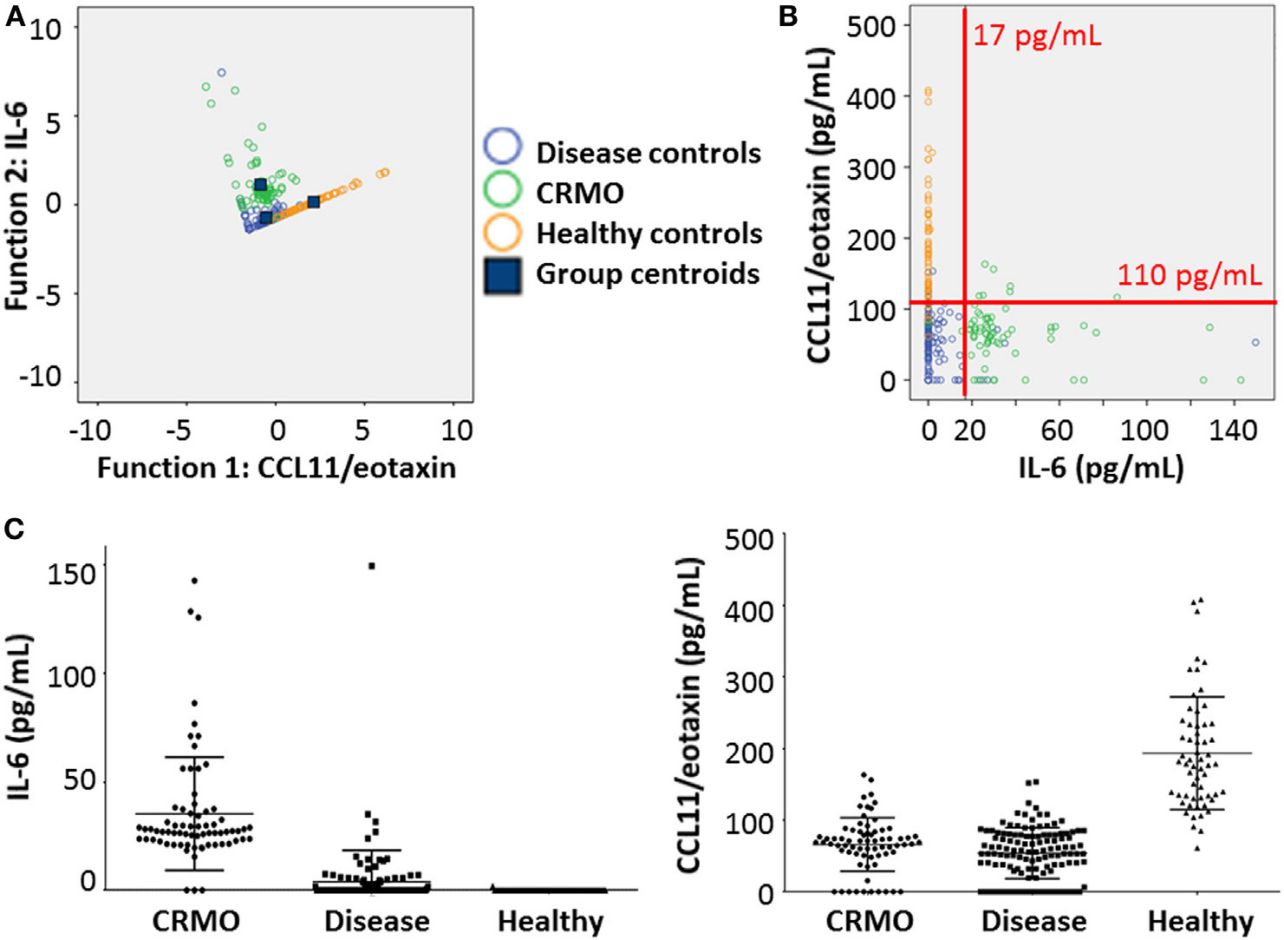
Validation of CCL11/eotaxin and IL-6 as serum biomarkers. **(A)** To test the applicability of serum IL-6 and CCL11/eotaxin for discriminating between healthy individuals, patients with chronic recurrent multifocal osteomyelitis (CRMO) or alternative diagnoses, a subsequent discriminant analysis between the disease groups and healthy controls was performed. **(B)** Serum levels of IL-6 and CCL11/eotaxin were plotted to test clustering within groups. Based on predictions from Figure 3, cutoff values were included as red lines. **(C)** Dot plots indicate ranges of IL-6 (left) and CCL11/eotaxin (right) values in sera from patient with alternative diagnoses, CRMO patients, or healthy controls.

To validate data, we performed canonical discriminant analysis, exclusively including the two remaining characteristics (Figure 4A; Data Supplement S5 in Supplementary Material). Indeed, serum samples from all three groups were discernable based on CCL11/eotaxin (function 1), and IL-6 (function 2). This was confirmed by scatter plot analysis (Figure 4B) showing clustering on measured values, based on disease group. Dot plot diagrams and subsequent ANOVA analysis with Bonferroni correction assured minimal or no overlap in the distribution of measured values between groups (Figure 4C; Data Supplement S6 in Supplementary Material).

### Correlation Analyses

To exclude potential correlation between patient characteristic and CCL11/eotaxin levels, correlation analysis was performed. Indeed, CCL11/eotaxin levels did not correlate with patient age (Figure S3A in Supplementary Material). Since patients with the included alternative diagnoses exhibited reduced CCL11/eotaxin serum levels when compared to controls, correlation between CrP and CCL11/eotaxin serum levels was tested. Of note, serum levels of CCL11/eotaxin correlated with CrP levels in patients with the included diagnoses (Figure S3B in Supplementary Material).

## Discussion

The autoinflammatory disorder CNO is a diagnosis of exclusion. Clinical overlaps with other inflammatory conditions, infections, and malignancies (acute leukemia and lymphoma) further complicate the situation (8, 9). Here, we measured 18 and identified 14 markers in sera from CRMO patients that were different between CRMO and at least one of the alternative diagnoses and/or healthy controls. In agreement with previous observations (10), elevated proteins in the serum of CRMO patients included cytokines (IL-6, RANKL/TRANCE), chemokines (CCL4/MIP-1b), and the soluble IL-2 receptor (sIL-2R).

### Cytokines

The anti-inflammatory molecule IL-1RA, the pro-inflammatory effector cytokines IL-6 and TNF-α, and the pro-inflammatory calcium binding molecule S100A8 (which is actually not considered a cytokine) were significantly elevated in sera from CRMO and/or other differential diagnoses when compared to healthy controls. We previously reported increased inflammasome activation and IL-1β expression in monocytes from CRMO patients. Activation of inflammatory caspase-1 is a central function of the NLRP3 inflammasome. IL-1β is a very unstable cytokine, which makes it virtually impossible to measure in routine serum samples. Here, IL-1RA levels were increased in sera from CRMO patients when compared to healthy controls, indirectly indicating increased inflammasome activation and IL-1β release. The post-translational regulator IL-1RA, however, directly reflects IL-1β release and may therefore be used as an indirect marker for IL-1β secretion (13). In a previous study (10), serum IL-1RA was not different between CRMO patients, JIA patients and healthy individuals. Differences between here presented data and previous observations may be explained with varying sensitivity between detection systems, since IL-1RA is usually considered a quite robust marker of inflammasome activation. In addition to IL-1β cleavage and activation, caspase-1 is also responsible for the activation of the pro-inflammatory cytokine IL-18 (14, 15). IL-18 is expressed by monocytes, lymphocytes, eosinophils, mast cells, and others. Main functions of IL-18 are monocyte activation, and the induction of pro-inflammatory cytokine expression (15, 16). Indeed, serum levels of IL-18 were increased in CRMO patients when compared to healthy controls. This, however, (likely due to large inter-individual variation) did not reach statistical significance.

The pro-inflammatory molecule S100A8 is secreted by monocytes and neutrophils, likely reflecting inflammasome activation and the induction of NF-κB signaling pathways (17). Indeed, S100A8 serum levels were increased in sera from CRMO patients when compared to healthy controls and individuals with leukemia. However, differences were small and inter-individual variation was relatively large.

Pro-inflammatory IL-6 is mainly produced by monocytes, fibroblasts, B and T cells, and plays a role in B and T cell activation and proliferation (18, 19). Inflammasome activation and resulting NF-κB signaling also plays a role in the regulation of IL-6 (16, 20). B and T cell-promoting effects of both cytokines IL-6 and IL-1β, may furthermore play a role during inflammatory responses in CRMO, since neutrophils and monocytes (which produce IL-6 and IL-1β) are the predominant cells in the acute phase of bone inflammation, while later stages are characterized by lymphocyte and plasma cell infiltration (2). Provided significant differences in serum protein levels, particularly IL-6 may be used to differentiate between CNO/CRMO, alternative included disorders and healthy controls. However, serum samples in (the few) patients with osteoarticular infections included in this study may have been collected in the post-acute phase. None of the included individuals were reported with high fever, highly inflamed joints, or massive CrP elevation. Thus, IL-6 may not be a good marker to differentiate between acute, febrile and highly inflammatory bacterial osteomyelitis and CRMO. This hypothesis is supported by the observation that IL-6 serum levels also normalize (quite rapidly) in CRMO patients in response to the introduction of treatment with NSAIDs (10).

The pro-inflammatory cytokine TNF-α is primarily produced by innate immune cells, osteoblasts, and smooth muscle cells. Key functions are monocyte activation, induction of osteoclast generation and activation, and the induction of cytokine expression (16, 20). In agreement with previous reports, serum TNF-α levels were increased in samples from CRMO patients (5, 7, 12). However, no differences could be detected between samples from CRMO patients and other included disorders. The osteoclast-promoting TNF-superfamily cytokine RANKL/TRANCE, however, showed a trend toward increased serum levels in CRMO/CNO as compared to healthy controls, which failed to reach statistical significance (16). RANKL/TRANCE serum levels in ANA positive oligoarticular JIA patients, however, were elevated when compared to CNO/CRMO, other differential diagnoses (acute leukemia, lymphoma), and healthy controls.

Some of our observations at first appear contradictory to previous studies. The immune-regulatory cytokine IL-10 fails to be expressed in monocytes from patients with CRMO (5, 7, 11, 12). In a previous serum biomarker study, we did not detect IL-10 in any of the disease or control groups, including CRMO (10). Here, we detected serum IL-10 in sera from all groups, including CRMO. This may be most likely due to higher sensitivity of the assay used in the present study as compared to previously applied assays (10). Indeed, IL-10 levels were slightly (but significantly) higher in CRMO patients when compared to healthy controls (but not other alternative diagnoses). This observation is not entirely surprising, since patients with other inflammatory diseases that are generally characterized by failure to express IL-10 from one or more cellular compartments may exhibit increased IL-10 serum levels when compared to healthy controls (e.g., oligoarticular JIA or systemic JIA) (21–26). Since CRMO is an inflammatory disorder and provided the fact that cells other than monocytes (e.g., lymphocytes, neutrophils, dendritic cells, eosinophils, etc., but also epithelia, stroma cells, etc.) express IL-10, serum IL-10 does not necessarily reflect gene expression from monocytes (27, 28). Furthermore, provided the large inter-individual variation in serum IL-10 levels together with the relatively small differences between groups, IL-10 was excluded from statistical analyses after the initial discriminant analysis.

In a previous study, IL-12 serum levels were different between patients with CNO vs. Crohn’s disease and JIA vs. healthy controls. Though statistically significant, differences were not very pronounced and showed overlap between groups (10). In contrast to these results, we failed to detect significant differences in serum IL-12p70 levels here. This may (as already briefly discussed for IL-10) likely be caused by the use of detection assays from different manufacturers in the two studies. Unfortunately, the use of different buffer systems and (probably even more importantly) different monoclonal detection antibodies significantly limit data comparability between many LUMINEX-based studies.

### Chemokines

In agreement with previous reports, two mostly monocyte-derived chemokines were elevated in the serum of CRMO patients as compared to healthy controls (CCL5/RANTES, CCL4/MIP-1β) (29). Also, monocytes from aseptic osteomyelitis-prone Pstpip2-deficient *cmo* and *lupo* mice exhibit elevated serum levels of CCL4/MIP-1β and IL-6 (30, 31).

Of note, in agreement with previous observations of our group and others, the eosinophil attracting chemokine CCL11/eotaxin was reduced in sera from patients with alternative diagnoses included in this study when compared to healthy controls (10, 32, 33). While CCL11/eotaxin levels vary between reports from different groups, they were reported reduced in patients with rheumatoid factor negative rheumatoid arthritis (32), and JIA patients with severe disease and radiographic progression (33). However, CCL11/eotaxin levels were reported increased in other inflammatory conditions including autoimmune disease (e.g., Sjögren’s syndrome) and infection (pulmonary tuberculosis) (34, 35). Regardless of variable results measuring CCL11/eotaxin levels in different forms of rheumatic disease, one feature reliably persists: JIA patients with high inflammatory activity and/or tendencies toward joint destruction, exhibit reduced levels of CCL11/eotaxin (33, 36). The question of whether variable pathomechanisms are responsible for inconsistent CCL11/eotaxin serum levels remains speculation and is beyond the scope of this report. Detection bias between samples from controls (from Würzburg) and patients (from Würzburg or Dresden) can be excluded, since samples from CRMO and JIA patients were collected at both institutions, and followed the same patterns with CCL11/eotaxin levels in the same range. Furthermore, trends were highly reproducible in two independent studies, and with assays from two manufacturers (10). Possible explanations for reduced CCL11/eotaxin serum levels in patients with here included alternative diagnoses and CRMO remain hypothetical and include “uptake,” recruitment to sites of inflammation, or cell-surface receptor binding of CCL11/eotaxin (32, 33). This may be supported by correlation between serum CrP and CCL11/eotaxin levels observed in the included individuals.

Provided significant differences in serum protein levels, eotaxin may be used to differentiate between healthy controls and all disease groups. Thus, CCL11/eotaxin may prove valuable to discriminate between patients with inflammatory conditions included here (including CRMO) and healthy individuals.

### Markers of Bone Metabolism

Provided potential effects of disturbed cytokine expression on the generation and activation of osteoclasts in CRMO, markers of bone remodeling were included in this study (12). Indeed, collagen Iα levels were increased in sera from CRMO patients as compared to healthy controls and patients with lymphoma, potentially indicating bone remodeling in CRMO and some other inflammatory conditions (37). Statistically significant differences in serum levels of osteoprotegerin were detected between patients with CRMO and lymphoma, while all other groups showed comparable results. A trend toward increased serum levels of SPARC/osteonectin in CRMO patients compared to healthy controls, likely due to large inter-individual variation, failed to reach statistical significance, while ANA positive oligoarticular JIA patients exhibited elevated levels when compared to CRMO patients.

Soluble IL-2 receptor concentrations can be measured in the serum, and correlate with disease activity and T cell activation in a number of inflammatory disorders, including CRMO, as well as malignancies (10, 38). Serum sIL-2R levels may be helpful for discriminating CRMO patients from individuals with acute leukemia and/or lymphoma, since both groups exhibited significantly higher serum levels (38). However, relevant overlap likely limits the applicability as reliable biomarker differentiation between these groups.

**Figure 5:**
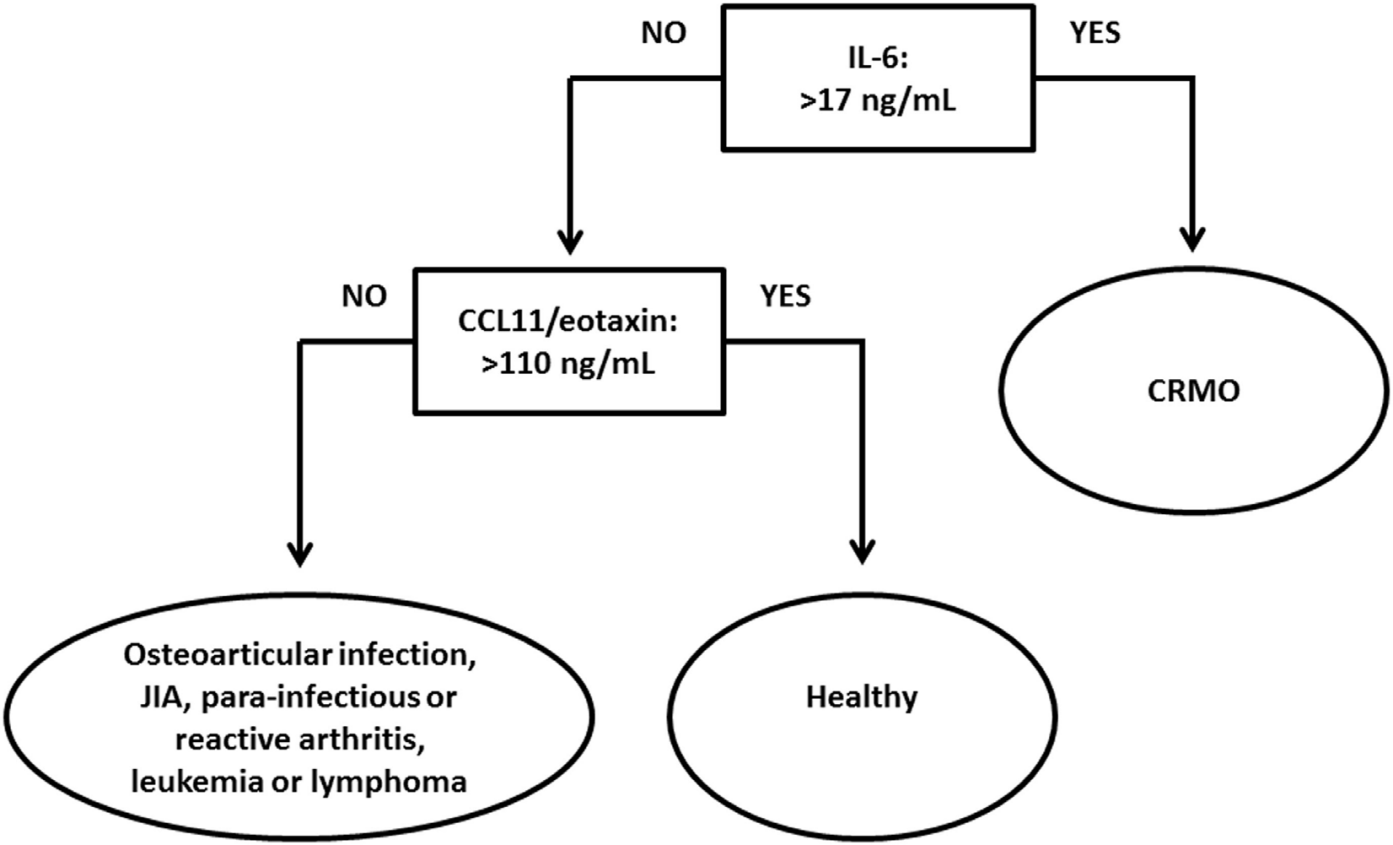
Proposed algorithm aiding the diagnosis of chronic recurrent multifocal osteomyelitis (CRMO). Based on observations in the presented serum protein analysis, CCL11/eotaxin may aid in the differentiation between healthy individuals and patients with CRMO or alternative diagnoses. In individuals with serum CCL11/eotaxin levels below 110 pg/mL (patients with CRMO or alternative diagnoses), IL-6 serum levels may help to discriminate between CRMO patients and individuals with here included alternative diagnoses.

Based on aforementioned results, discriminant analyses allowed discernment between CRMO, alternative diagnoses, and healthy individuals. Particularly the pro-inflammatory cytokine IL-6, the chemokines CCL11/eotaxin and CCL5/RANTES, the bone remodeling marker collagen Iα, and sIL-2R promise potential as disease biomarkers for CRMO. Indeed, in the investigated cohort, restriction of biomarkers to CCL11/eotaxin and IL-6 still allowed discrimination between CRMO, important alternative diagnoses, and healthy individuals with a high sensitivity (all >92%) and specificity (all >87%). Based on clinical experience and the relatively low cutoff for IL-6 serum levels (17 pg/mL), screening of CCL11/eotaxin serum levels, followed by IL-6 serum level determination may be more reasonable. Taken together, we for the first time report potentially reliable and easily accessible parameters that may find their way into routine diagnosis and care of patients with CRMO allowing exclusion of differential diagnoses (Figure 5). Correlation between age and CCL11/eotaxin levels were (largely) excluded as potential bias. Furthermore, in all groups, CCL11/eotaxin correlated with CrP levels.

The inclusion of additional differential diagnoses and further serum markers, enabling discrimination between differential diagnoses, the presented study significantly extends beyond the aims and results of previous studies (10). However, at least at this stage, proposed serum markers do certainly not replace the exclusion of differential diagnoses. Presented results require confirmation in larger unrelated cohorts. Prospectively collected bio samples from patients with CNO/CRMO, various forms of JIA, and other inflammatory differential diagnoses together with detailed information on clinical activity will allow further evaluation of the applicability of serum IL-6 as discriminator between CRMO and differential diagnoses. A limitation of the presented study is the lack of clinical information on disease activity in included inflammatory conditions. Bio sample collection and precise documentation of clinical data during *consensus treatment plan* and *treat to target* efforts in large cohorts, as currently planned by the Childhood Arthritis and Rheumatology Research Alliance (CARRA) and the German Society of Pediatric Rheumatology (GKJR) may provide necessary patient numbers and clinical data sets to tackle this task.

## Conclusion

Two serum biomarkers (CCL11/eotaxin, IL-6) discriminate between CRMO, alternative diagnoses (acute leukemia, lymphoma, JIA, osteoarticular infections, and reactive or para-infectious arthritis), and healthy controls. CCL11/eotaxin may be useful for discerning between healthy individuals and patients with (non-allergic) inflammatory conditions. Confirmation in large and unrelated cohorts is required. However, results require to be interpreted with caution secondary to limited sample size, lacking information on disease activity in JIA patients, and the current absence of independent control cohorts.

## Ethics Statement

The Ethics Committees of the University of Technology Dresden, and the University of Würzburg approved collection and use of these samples. Serum samples from patients with other diagnoses were collected during routine care and stored in the bio-repository for potential additional diagnostic tests. Retrospective sample identification, collection, and use of anonymized samples were approved by the Ethics Committe of the University of Technology Dresden. Since ethics approval required complete anonymization of all samples, retrospective analyses of JIA disease activity or long-term outcomes was impossible.

## Author Contributions

CH, SH, HM, HG, UR, and MS designed the experiments. SH, FB, and CL performed the experiments. All authors contributed to data interpretation. CH, SH, and HG wrote the manuscript. All authors edited and agreed to the final version of the manuscript.

## Conflict of Interest Statement

The authors declare that the research was conducted in the absence of any commercial or financial relationships that could be construed as a potential conflict of interest.

## Supplementary Material

Figure S1 Serum inflammation markers in chronic recurrent multifocal osteomyelitis (CRMO) and alternative diagnoses. 18 serum inflammation markers were determined in samples from 71 CRMO patients, 11 patients with osteoarticular infections, 62 patients with various forms of juvenile idiopathic arthritis (JIA), 7 patients with para-infectious or reactive arthritis, and 43 patients with acute leukemia or lymphoma, as well as 59 healthy individuals using a custom multiplex assay (R&D Systems) on the Luminex^®^ 200™ platform. For the displayed inflammation markers, serum levels were detectable and significantly different for at least two of the groups. *p* values from univariate ANOVA tests applying Bonferroni correction and pairwise comparison are given. Adjusted *p* values of <0.05 were considered statistically significant.

Figure S2 Identification of optimal biomarkers and cutoff prediction. Receiver operating characteristic (ROC) analysis delivered CCL11/eotaxin as a promising candidate in the search for biomarkers differentiating between all included conditions and healthy controls, and IL-6 as a potential biomarker discriminating between chronic recurrent multifocal osteomyelitis (CRMO) and all others. Furthermore, ROC analysis suggested CCL5/RANTES, collagen Iα, and sIL-2R as potential diagnostic biomarkers.

Figure S3 Potential correlation between CCL11/eotaxin and other variables. **(A)** CCL11/eotaxin levels do not correlate with age. **(B)** CCL11/eotaxin correlates with C-reactive protein (CrP) levels in chronic recurrent multifocal osteomyelitis (CRMO) patients, and disease controls.

Data Supplement S1 Demographic information on included patients.

Data Supplement S2 Univariate ANOVA with Bonferroni correction and pairwise comparison [chronic recurrent multifocal osteomyelitis (CRMO) vs. controls vs. alternative diagnoses].

Data Supplement S3 Standardized canonical discriminant analysis.

Data Supplement S4 Sensitivity and specificity of predictive approach.

Data Supplement S5 Standardized canonical discriminant analysis with two core variables.

Data Supplement S6 Univariate ANOVA with Bonferroni correction and pairwise comparison [chronic recurrent multifocal osteomyelitis (CRMO) vs. controls vs. pooled alternative diagnoses].

## Abbreviations

CCL, chemokine (C-C motif) ligand; CNO, chronic nonbacterial osteomyelitis; CRMO, chronic recurrent multifocal osteomyelitis; CrP, C-reactive protein; Eotaxin, C-C motif chemokine 11, also known as eosinophil chemotactic protein and eotaxin-1; IL, interleukin; IL-1RA, IL-1 receptor antagonist; JIA, juvenile idiopathic arthritis; MIP, macrophage inflammatory protein; RANKL, receptor activator of nuclear factor kappa-B ligand; RANTES, regulated on activation, normal T cell expressed and secreted protein, also known as chemokine (C-C motif) ligand 5 (CCL5); sIL-2R, soluble IL-2 receptor; sJIA, systemic juvenile idiopathic arthritis; SPARC, secreted protein acidic and rich in cysteine; TNF-α, tumor necrosis factor α; TRANCE, TNF-related activation-induced cytokine.

## References

[1] Hedrich CM Hahn G Girschick HJ Morbach H. A clinical and pathomechanistic profile of chronic nonbacterial osteomyelitis/chronic recurrent multifocal osteomyelitis and challenges facing the field. Expert Rev Clin Immunol (2013) 9(9):845–54. 10.1586/1744666X.2013.824670 24070048

[2] Hedrich CM Hofmann SR Pablik J Morbach H Girschick HJ Autoinflammatory bone disorders with special focus on chronic recurrent multifocal osteomyelitis (CRMO). Pediatr Rheumatol Online J (2013) 11(1):47 10.1186/1546-0096-11-47 24359092 PMC3881012

[3] Stern SM Ferguson PJ. Autoinflammatory bone diseases. Rheum Dis Clin North Am (2013) 39(4):735–49. 10.1016/j.rdc.2013.05.002 24182852 PMC3823499

[4] Hofmann SR Roesen-Wolff A Hahn G Hedrich CM. Update: cytokine dysregulation in chronic nonbacterial osteomyelitis (CNO). Int J Rheumatol (2012) 2012:310206. 10.1155/2012/310206 22685464 PMC3364585

[5] Hofmann SR Morbach H Schwarz T Rosen-Wolff A Girschick HJ Hedrich CM. Attenuated TLR4/MAPK signaling in monocytes from patients with CRMO results in impaired IL-10 expression. Clin Immunol (2012) 145(1):69–76. 10.1016/j.clim.2012.07.012 22940633

[6] Hamel J Paul D Gahr M Hedrich CM Pilot study: possible association of IL10 promoter polymorphisms with CRMO. Rheumatol Int (2012) 32(2):555–6. 10.1007/s00296-010-1768-8 21240493

[7] Hofmann SR Schwarz T Moller JC Morbach H Schnabel A Rosen-Wolff A Chronic non-bacterial osteomyelitis is associated with impaired Sp1 signaling, reduced IL10 promoter phosphorylation, and reduced myeloid IL-10 expression. Clin Immunol (2011) 141(3):317–27. 10.1016/j.clim.2011.08.012 21925952

[8] Jansson AF Muller TH Gliera L Ankerst DP Wintergerst U Belohradsky BH Clinical score for nonbacterial osteitis in children and adults. Arthritis Rheum (2009) 60(4):1152–9. 10.1002/art.24402 19333943

[9] Girschick HJ Zimmer C Klaus G Darge K Dick A Morbach H. Chronic recurrent multifocal osteomyelitis: what is it and how should it be treated? Nat Clin Pract Rheumatol (2007) 3(12):733–8. 10.1038/ncprheum0653 18037933

[10] Hofmann SR Kubasch AS Range U Laass MW Morbach H Girschick HJ Serum biomarkers for the diagnosis and monitoring of chronic recurrent multifocal osteomyelitis (CRMO). Rheumatol Int (2016) 36(6):769–79. 10.1007/s00296-016-3466-7 27000045

[11] Hofmann SR Kubasch AS Ioannidis C Rosen-Wolff A Girschick HJ Morbach H Altered expression of IL-10 family cytokines in monocytes from CRMO patients result in enhanced IL-1beta expression and release. Clin Immunol (2015) 161(2):300–7. 10.1016/j.clim.2015.09.013 26404542

[12] Hofmann SR Schnabel A Rosen-Wolff A Morbach H Girschick HJ Hedrich CM. Chronic nonbacterial osteomyelitis: pathophysiological concepts and current treatment strategies. J Rheumatol (2016) 43(11):1956–64. 10.3899/jrheum.160256 27585682

[13] Bruserud O Aasen I Akselsen PE Bergheim J Rasmussen G Nesthus I. Interleukin 1 receptor antagonist (IL1RA) in acute leukaemia: IL1RA is both secreted spontaneously by myelogenous leukaemia blasts and is a part of the acute phase reaction in patients with chemotherapy-induced leucopenia. Eur J Haematol (1996) 57(1):87–95. 10.1111/j.1600-0609.1996.tb00495.x 8698137

[14] Winkler S Hedrich CM Rosen-Wolff A. [Caspase-1 regulates autoinflammation in rheumatic diseases]. Z Rheumatol (2016) 75(3):265–75. 10.1007/s00393-016-0077-3 27034076

[15] Winkler S Rosen-Wolff A. Caspase-1: an integral regulator of innate immunity. Semin Immunopathol (2015) 37(4):419–27. 10.1007/s00281-015-0494-4 26059719

[16] McInnes IB Cytokines. 10th ed In: Firesetin GS Budd RC Sherine EG McInnes IB O’Dell SR, editors. Kelley and Firestein’s Textbook of Rheumatology. Philadelphia, PA: Elsevier (2017). p. 396–407.

[17] Kessel C Holzinger D Foell D. Phagocyte-derived S100 proteins in autoinflammation: putative role in pathogenesis and usefulness as biomarkers. Clin Immunol (2013) 147(3):229–41. 10.1016/j.clim.2012.11.008 23269200

[18] Eulenfeld R Dittrich A Khouri C Muller PJ Mutze B Wolf A Interleukin-6 signalling: more than Jaks and STATs. Eur J Cell Biol (2012) 91(6–7):486–95. 10.1016/j.ejcb.2011.09.010 22138086

[19] McInnes IB Cytokines. 9th ed In: Firestein GS Budd RC Harris ED Jr, editors. Kelley’s Textbook of Rheumatology. Philadelphia, PA: Elsevier (2013). p. 367–77.

[20] Bruck N Schnabel A Hedrich CM. Current understanding of the pathophysiology of systemic juvenile idiopathic arthritis (sJIA) and target-directed therapeutic approaches. Clin Immunol (2015) 159(1):72–83. 10.1016/j.clim.2015.04.018 25956529

[21] Brescia AC Simonds MM Sullivan KE Rose CD Secretion of pro-inflammatory cytokines and chemokines and loss of regulatory signals by fibroblast-like synoviocytes in juvenile idiopathic arthritis. Proteomics Clin Appl (2017) 11:5–6. 10.1002/prca.201600088 PMC6084365 28012239

[22] Crawley E Kon S Woo P. Hereditary predisposition to low interleukin-10 production in children with extended oligoarticular juvenile idiopathic arthritis. Rheumatology (Oxford) (2001) 40(5):574–8. 10.1093/rheumatology/40.5.574 11371669

[23] Fife MS Gutierrez A Ogilvie EM Stock CJ Samuel JM Thomson W Novel IL10 gene family associations with systemic juvenile idiopathic arthritis. Arthritis Res Ther (2006) 8(5):R148. 10.1186/ar2041 16959027 PMC1779449

[24] Moller JC Paul D Ganser G Range U Gahr M Kelsch R IL10 promoter polymorphisms are associated with systemic onset juvenile idiopathic arthritis (SoJIA). Clin Exp Rheumatol (2010) 28(6):912–8. 21205466

[25] Shahin AA Shaker OG Kamal N Hafez HA Gaber W Shahin HA. Circulating interleukin-6, soluble interleukin-2 receptors, tumor necrosis factor alpha, and interleukin-10 levels in juvenile chronic arthritis: correlations with soft tissue vascularity assessed by power Doppler sonography. Rheumatol Int (2002) 22(2):84–8. 10.1007/s00296-002-0191-1 12070682

[26] Muller K Herner EB Stagg A Bendtzen K Woo P. Inflammatory cytokines and cytokine antagonists in whole blood cultures of patients with systemic juvenile chronic arthritis. Br J Rheumatol (1998) 37(5):562–9. 10.1093/rheumatology/37.5.562 9651086

[27] Hedrich CM Bream JH. Cell type-specific regulation of IL-10 expression in inflammation and disease. Immunol Res (2010) 47(1–3):185–206. 10.1007/s12026-009-8150-5 20087682 PMC2892196

[28] Hofmann SR Rosen-Wolff A Tsokos GC Hedrich CM Biological properties and regulation of IL-10 related cytokines and their contribution to autoimmune disease and tissue injury. Clin Immunol (2012) 143(2):116–27. 10.1016/j.clim.2012.02.005 22459704

[29] Griffith JW Sokol CL Luster AD. Chemokines and chemokine receptors: positioning cells for host defense and immunity. Annu Rev Immunol (2014) 32:659–702. 10.1146/annurev-immunol-032713-120145 24655300

[30] Chitu V Ferguson PJ de Bruijn R Schlueter AJ Ochoa LA Waldschmidt TJ Primed innate immunity leads to autoinflammatory disease in PSTPIP2-deficient cmo mice. Blood (2009) 114(12):2497–505. 10.1182/blood-2009-02-204925 19608749 PMC2746474

[31] Grosse J Chitu V Marquardt A Hanke P Schmittwolf C Zeitlmann L Mutation of mouse Mayp/Pstpip2 causes a macrophage autoinflammatory disease. Blood (2006) 107(8):3350–8. 10.1182/blood-2005-09-3556 16397132 PMC1895761

[32] Chalan P Bijzet J van den Berg A Kluiver J Kroesen BJ Boots AM Analysis of serum immune markers in seropositive and seronegative rheumatoid arthritis and in high-risk seropositive arthralgia patients. Sci Rep (2016) 6:26021. 10.1038/srep26021 27189045 PMC4870704

[33] Syversen SW Goll GL Haavardsholm EA Boyesen P Lea T Kvien TK. A high serum level of eotaxin (CCL 11) is associated with less radiographic progression in early rheumatoid arthritis patients. Arthritis Res Ther (2008) 10(2):R28. 10.1186/ar2381 18312691 PMC2453772

[34] Sharifabadi AR Hassanshahi G Ghalebi SR Arababadi MK Khorramdelazad H Zainodini N All eotaxins CCL11, CCL24 and CCL26 are increased but to various extents in pulmonary tuberculosis patients. Clin Lab (2014) 60(1):93–7. 10.7754/Clin.Lab.2013.121231 24600981

[35] Nocturne G Seror R Fogel O Belkhir R Boudaoud S Saraux A CXCL13 and CCL11 serum levels and lymphoma and disease activity in primary Sjogren’s syndrome. Arthritis Rheumatol (2015) 67(12):3226–33. 10.1002/art.39315 26359802

[36] de Jager W Hoppenreijs EP Wulffraat NM Wedderburn LR Kuis W Prakken BJ. Blood and synovial fluid cytokine signatures in patients with juvenile idiopathic arthritis: a cross-sectional study. Ann Rheum Dis (2007) 66(5):589–98. 10.1136/ard.2006.061853 17170049 PMC1954617

[37] Eriksen EF Charles P Melsen F Mosekilde L Risteli L Risteli J. Serum markers of type I collagen formation and degradation in metabolic bone disease: correlation with bone histomorphometry. J Bone Miner Res (1993) 8(2):127–32. 10.1002/jbmr.5650080202 8442431

[38] Sakai A Yoshida N. The role of tumor-associated macrophages on serum soluble IL-2R levels in B-cell lymphomas. J Clin Exp Hematop (2014) 54(1):49–57. 10.3960/jslrt.54.49 24942946

