# Supplementary figures and images for: Serum Interleukin-6 and CCL11/Eotaxin May Be Suitable Biomarkers for the Diagnosis of Chronic Nonbacterial Osteomyelitis
Source: Front Pediatr. 2017 Dec 1;5:256. doi: 10.3389/fped.2017.00256 (PMC5716982; doi:10.3389/fped.2017.00256)

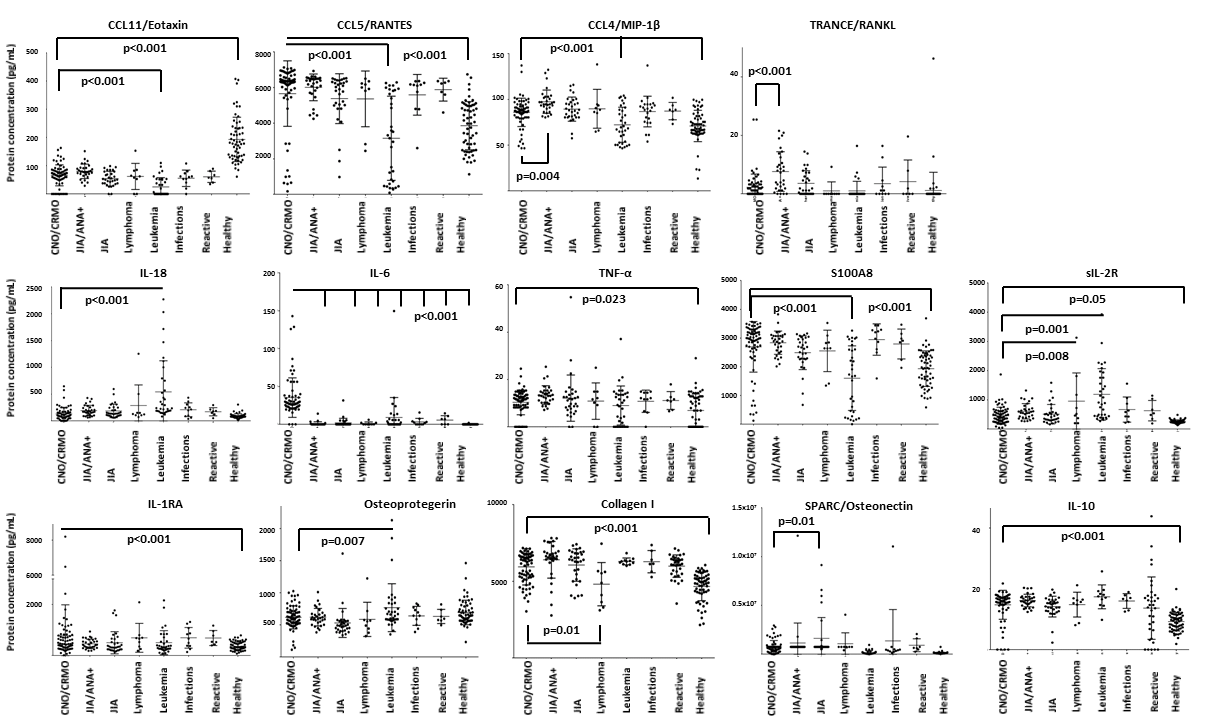

Supplement: Figure S1 — Serum inflammation markers in chronic recurrent multifocal osteomyelitis (CRMO) and alternative diagnoses. 18 serum inflammation markers were determined in samples from 71 CRMO patients, 11 patients with osteoarticular infections, 62 patients with various forms of juvenile idiopathic arthritis (JIA), 7 patients with para-infectious or reactive arthritis, and 43 patients with acute leukemia or lymphoma, as well as 59 healthy individuals using a custom multiplex assay (R&D Systems) on the Luminex® 200™ platform. For the displayed inflammation markers, serum levels were detectable and significantly different for at least two of the groups. p values from univariate ANOVA tests applying Bonferroni correction and pairwise comparison are given. Adjusted p values of <0.05 were considered statistically significant. [file image_1.tif]

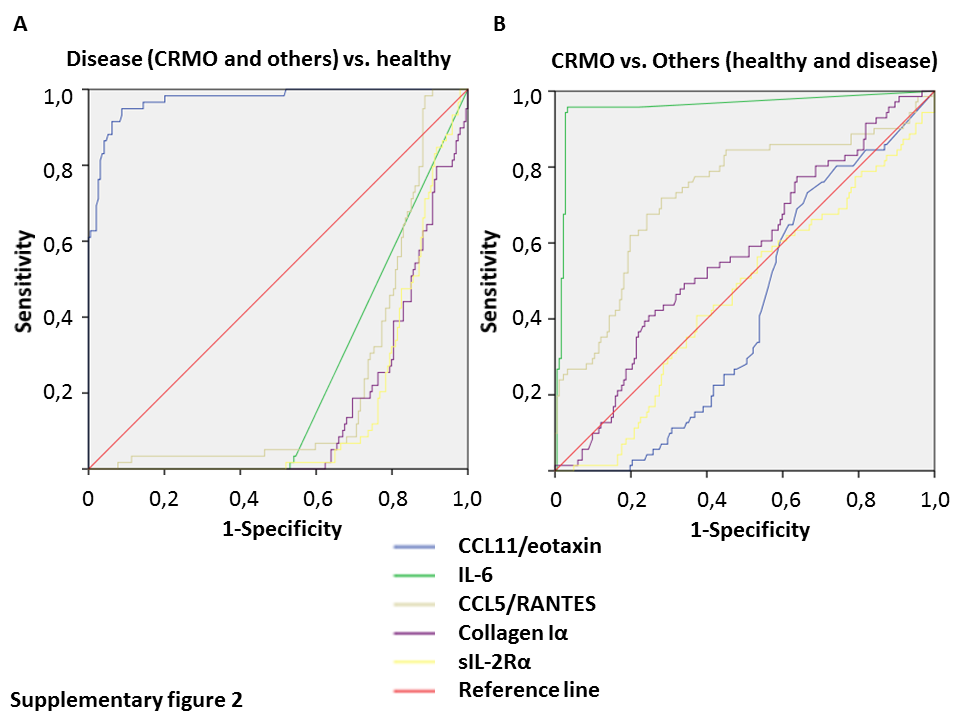

Supplement: Figure S2 — Identification of optimal biomarkers and cutoff prediction. Receiver operating characteristic (ROC) analysis delivered CCL11/eotaxin as a promising candidate in the search for biomarkers differentiating between all included conditions and healthy controls, and IL-6 as a potential biomarker discriminating between chronic recurrent multifocal osteomyelitis (CRMO) and all others. Furthermore, ROC analysis suggested CCL5/RANTES, collagen Iα, and sIL-2R as potential diagnostic biomarkers. [file image_2.tif]

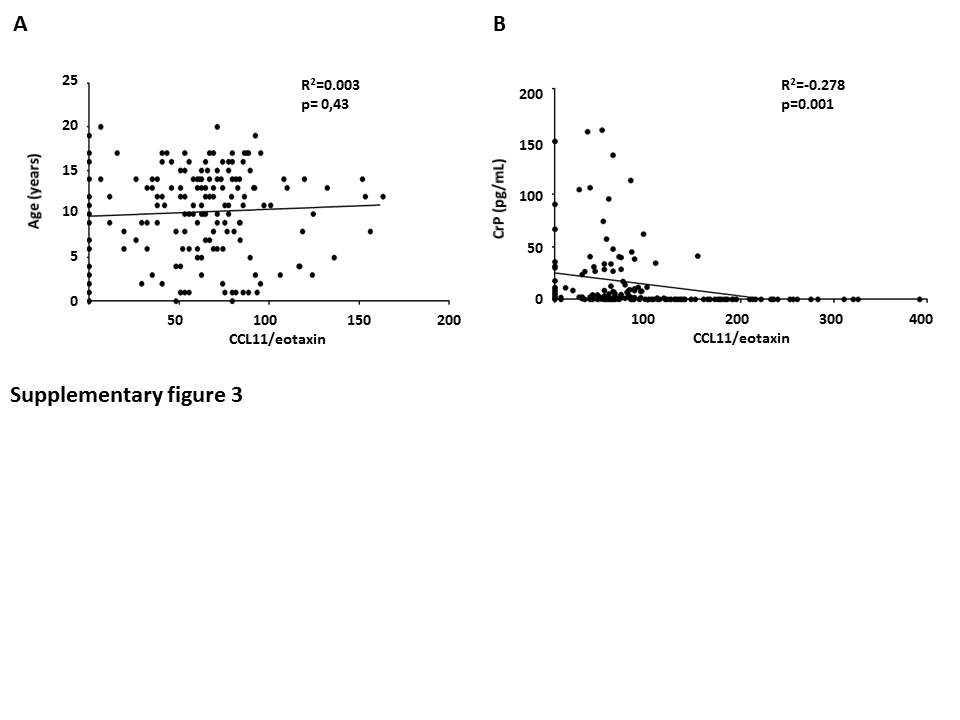

Supplement: Figure S3 — Potential correlation between CCL11/eotaxin and other variables. (A) CCL11/eotaxin levels do not correlate with age. (B) CCL11/eotaxin correlates with C-reactive protein (CrP) levels in chronic recurrent multifocal osteomyelitis (CRMO) patients, and disease controls. [file image_3.tif]
